# International Nephrology Masterclass in Chronic Kidney Disease: Rationale, Summary, and Future Perspectives

**DOI:** 10.3390/life14121668

**Published:** 2024-12-17

**Authors:** Francesco Pesce, Maria Vadalà, Edgar Almeida, Beatriz Fernandez, Denis Fouque, Jolanta Malyszko, Kai Schmidt-Ott, Peter Stenvinkel, David C. Wheeler, Samuel Seidu, Ana Cebrian, Nikolay Dimov, Marta Blanco Pardo, Ieva Ziedina, Nayaf Habashi, Joaquin Manrique, Sofia Homem De Melo Marques, Marco Antonio Vaca Gallardo, Larisa Shehaj, Milena Krasimirova Nikolova Vlahova, Luis Mendonça, Sara Ksiazek, Pierangelo Veltri, Giuseppe Pezzi, Gemma Patella, Greta Borelli, Michele Provenzano, Loreto Gesualdo

**Affiliations:** 1 Department of Translational Medicine and Surgery, Università Cattolica del Sacro Cuore, 00168 Rome, Italy; f.pesce81@gmail.com; 2 Division of Renal Medicine, Ospedale Isola Tiberina-Gemelli, 00816 Rome, Italy; mary.vadala@gmail.com; 3 Hospital da Luz, 1500-650 Lisboa, Portugal; edgar.almeida@hbeatrizangelo.pt; 4 Hospital Fundaciòn Jimenez-Diaz, 28040 Madrid, Spain; bfernandez@fjd.es; 5 University Claude Bernard Lyon, 69100 Villeurbanne, France; 6 Medical University Warsaw, 02-091 Warszawa, Poland; jolmal@poczta.onet.pl; 7 Department of Nephrology and Hypertension, Hannover Medical School, 30625 Hannover, Germany; schmidt-ott.kai@mh-hannover.de; 8 Karolinska Hospital, 171 76 Stockholm, Sweden; peter.stenvinkel@ki.se; 9 University College London, London WC1E 6BT, UK; d.wheeler@ucl.ac.uk; 10 Leicester City Clinical Commissioning Group, Leicester LE1 6NB, UK; sis11@leicester.ac.uk; 11 Cartagena Casco Health Center, 30201 Murcia, Spain; anicebrian@gmail.com; 12 Nephrology Clinic, University Hospital “Sv. Georgi”, 4002 Plovdiv, Bulgaria; nikolai.r.dimov@gmail.com; 13 División of Nephrology, A Coruña University Hospital, 15006 A Coruña, Spain; martabp94@gmail.com; 14 Riga Stradins University, LV-1007 Riga, Latvia; ieva.ziedina@rsu.lv; 15 Department of Nephrology, HaEmeq Hospital Afula, Afula 1834111, Israel; habashinayaf@gmail.com; 16 Servicio de Nefrología, Complejo Hospitalario de Navarra, 31008 Pamplona, Spain; jmanriquees@gmail.com; 17 Hemodialysis Clinic Hospital Agostinho Ribeiro, 4610-106 Felgueiras, Portugal; sofiahomemdemelo@gmail.com; 18 Servicio de Nefrología, Hospital Universitario La Paz, 28046 Madrid, Spain; marco.vaca.g@gmail.com; 19 Department of Nephrology, Faculty of Medicine, Bezmialem Vakif University, Istanbul 34093, Türkiye; lorishehaj1@gmail.com; 20 University Hospital St. Ivan Rilski, 1000 Sofia, Bulgaria; milena_i_dani@abv.bg; 21 Unit of Cardiovascular Research and Development—Unic@RISE, Department of Surgery and Physiology, Faculty of Medicine, University of Porto, 4200-319 Porto, Portugal; luiscfmendonca@gmail.com; 22 6th Medical Department of Internal Medicine with Nephrology & Dialysis, Clinic Ottakring, 1160 Vienna, Austria; sara.ksiazek@hotmail.com; 23 Department of Computer Science, Modeling, Electronics and Systems Engineering, University of Calabria, 87036 Rende, Italy; pierangelo.veltri@dimes.unical.it; 24 Department of Medical and Surgical Sciences, University of Catanzaro, 88100 Catanzaro, Italy; giuseppe.pezzi@unicz.it; 25 Department of Nephrology, Azienda Sanitaria Provinciale, 87100 Cosenza, Italy; gemmapatella@hotmail.it; 26 Nephrology, Dialysis and Renal Transplant Unit, IRCSS-Azienda Ospedaliero-Universitaria di Bologna, 40138 Bologna, Italy; greta.borelli@studio.unibo.it; 27 Department of Pharmacy, Health and Nutritional Sciences, University of Calabria, 87036 Rende, Italy; 28 Department of Emergency and Organ Transplantation, University of Bari Aldo Moro, 70121 Bari, Italy; loretoge60@gmail.com

**Keywords:** masterclass, nephrology, CKD, kidney, disease, educational, gender, diabetes, hypertension, genetics

## Abstract

Chronic kidney disease (CKD) is a progressive condition that affects more than 10% of the population worldwide, accounting for more than 843 million (M) individuals. The prevalence of CKD (844 M patients) is higher than that of diabetes mellitus (422 M patients), cancer (42 M patients), and HIV (37 M patients), but people are often less aware of it. Global expert groups predict reductions in the nephrology workforce in the next decade, with a declining interest in nephrology careers. Over time, KDIGO guidelines have also focused on topics related to the prevention or management of CKD patients in real-life settings. On these premises, a new educational program with international experts in the field of nephrology took place from November 2022 until March 2023 in Milan, Italy. This multinational masterclass provided an educational platform providing unbiased education on diagnosis and treatment by sharing the most recent research data on CKD and comorbidities, therefore creating a snowball effect to increase the implementation of best practices worldwide, using examples from ‘real-life’ patient outcomes. This paper provides an overview of the International Nephrology Masterclass (INM) concept, summarizing the key lectures and discussions, and giving an outline of future key developments.

## 1. Introduction

Chronic kidney disease (CKD) is a silent and very frequent non-communicable disease (NCD), that, according to the Global Burden of Diseases, Injuries and Risk Factors Study, is expected to become the fifth cause of death worldwide by 2040 [1]. It represents a progressive condition that affects > 10% of the global population, amounting to 843.6 million (M) individuals, and has emerged as a significant public health crisis [2,3]. The incidence of CKD has risen due to the increased prevalence of risk factors, making it a major global health concern. The impact of CKD is classified as the 12th and 17th leading cause of death and disability worldwide, respectively [4]. It is characterized by a gradual decline in renal function, often linked to risk factors such as diabetes, hypertension, poor diet, and aging (most deaths from CKD occur in people over 65 years of age) [5]. The prevalence of kidney disease (844 M patients) is higher than that of diabetes mellitus (422 M patients), cancer (42 M patients), and HIV (37 M patients); however, there is less public awareness [6]. A low estimated glomerular filtration rate (eGFR), the main marker of kidney function, is identified as a significant contributor to global mortality and disability. Individuals with CKD face an elevated risk of kidney failure, cardiovascular disease, and premature death, emphasizing the urgency of addressing this health issue. CKD also poses a substantial financial burden, accounting for a notable percentage of annual healthcare expenditures in high-income countries [7]. This is particularly noteworthy considering that patients with kidney failure (KF, the most severe stage of CKD) constitute a small fraction of the total population. Moreover, lower socioeconomic status increases one’s risk of KF [8]. Prevention and the implementation of strategies for the early diagnosis, therapy and follow-up of CKD are a key challenge for the main healthcare systems. Global expert groups predict reductions in the nephrology workforce over the next decade, with potentially serious implications. This is identified as a new arising nephrological phenomenon, called Acute Fellowship Insufficiency (AFI), resulting in a declining interest in nephrology careers with 51% of programs going unfilled, as evidenced by the American Society of Nephrology (ASN) in 2016. Cost-effective and high-quality management of CKD at the population level is a healthcare priority. Although CKD is one of the most frequent NCDs, we will be facing a reduction in the nephrology workforce over the next decade, with potentially serious implications. Moreover, real-life data show that most renal patients do not follow guidelines and have a very poor adherence to treatment [9,10]. Therefore, the International Nephrology Masterclass (INM) was created to fill this gap and inform many clinicians, which will further spread the best practices at national and regional levels. It was organized for the first time from November 2022 to March 2023, with the purpose of bringing focus to CKD awareness, early diagnosis, complications, new therapeutic approaches, and training young, talented renal fellows on CKD.

**Figure 1:**
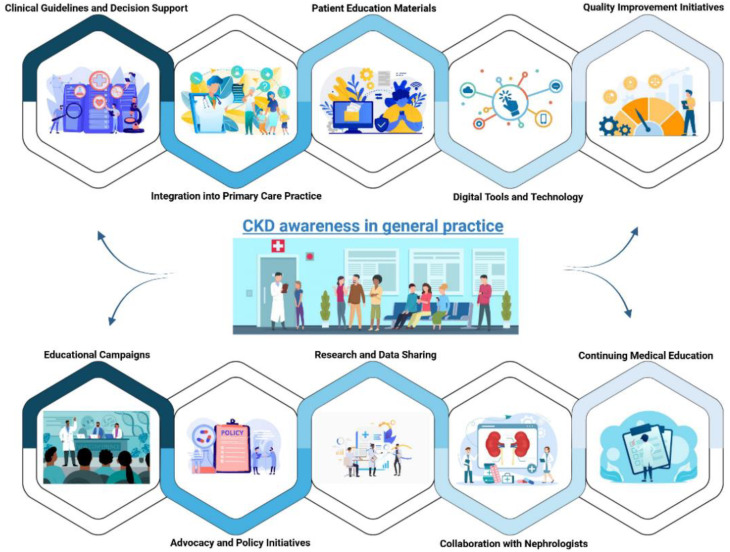
Models to increase awareness of chronic kidney disease.

This new educational format aimed to increase the appeal of a career in nephrology by fostering a community of motivated nephrologists with expertise on CKD and a willingness to spread awareness about the importance of being a nephrologist among other trainees. To achieve this, the project used modern and cutting-edge digital communication technologies such as a TV studio, news, and talk-show format, along with an online platform, which served as the project’s main hub. Additionally, three live streaming events were held in 2022, culminating in a final in-person event on 17/18 March 2023, at Cascina Triulza, MIND, Milan, Italy. This first INM was attended by over 90 nephrologists from 21 European countries as well as those engaging online (Figure 1). The participating universities included University Claude Bernard Lyon, Bari University, Medical University Warsaw, and University College London. This educational program was designed to update the participants on the latest data from clinical trials as well as recent real-life evidence. The aim of this paper is to describe in detail the broad educational program and practical workshops on CKD that took place during the INM, with a focus on awareness, diagnostics, special conditions and cutting-edge science, training the next generations of leaders in CKD management, and creating a community of motivated specialists with expertise on CKD who are able to convey the importance of being a nephrologist to other trainees.

## 2. The Rationale Behind the Masterclass

The topics addressed in the educational path included the identification of patients at risk of CKD; CKD complications (such as hypertension, anemia, bone and mineral disorders, and metabolic acidosis); diagnosis and treatment in primary care; and, finally, an in-depth analysis of the International Society of Nephrology (ISN)/KDIGO guidelines. Patients at high risk for CKD are especially those with diabetes and hypertension, accounting for more than half of all clinical cases (42% and 18%, respectively), so an improvement in population screening and diagnosis is needed, as showed by different international researchers [11,12,13,14,15]. Chronic kidney disease is also characterized by several complications including hypertension, anemia, hyperkalemia, dyslipidemia. During the masterclass, we discussed the results of the Systolic Blood Pressure Intervention Trial (SPRINT) trial, which enrolled 9361 American people > 50 yrs with a systolic blood pressure of 130 mm Hg or higher and an increased cardiovascular risk, but without diabetes, who were assigned to a systolic blood pressure target of less than 120 mmHg (intensive treatment) or a target of less than 140 mmHg (standard treatment) [16]. All-cause mortality was also significantly lower in the intensive-treatment group. Serious adverse events, including hypotension, syncope, and acute kidney injury or failure were higher in the intensive-treatment group than in the standard-treatment group [16,17]. Another current complication of stage 3b-4 CKD is hyperkalemia. As is known, due to the chronicity of hyperkalemia CKD patients can tolerate higher levels of potassium without being symptomatic. While hyperkalemia should still be treated, when potassium excretion in declining kidney function kidneys does not work, the only alternative becomes the improvement of the excretion of potassium through the gastrointestinal tract. This can be achieved by implementing a potassium-restricted diet, adding loop diuretics, adding potassium binders, treatment of an underlying metabolic acidosis, or the reduction/stop of renin–angiotensin–aldosterone system (RAAS) blockers [18].

Anemia represents another severe CKD complication, which until the 1990s was only possible to be treated with transfusions which, in turn, caused hypertension and stroke. The emergence of erythropoiesis-stimulating agents (ESAs) (1990s), as well as intravenous (iv) and oral iron (2000s) and, in recent years, HIF stabilizers (2020s), changed the landscape of anemia treatment in CKD patients substantially [19,20]. Another complication starting in CKD stage 3b-4 is mineral and bone disorder (CKD-MBD), which involves abnormalities in bone and mineral metabolism and/or extra-skeletal calcification [21]. In this case, the nephrologist should treat these patients with a calcimimetic, a drug that mimics the effects of calcium on the parathyroid glands and may trick the parathyroid glands into releasing less parathyroid hormone [22]. The last CKD complication addressed in the masterclass was dyslipidemia. We discussed the results of the Study of Heart and Renal Protection (SHARP) trial, which enrolled 9270 CKD patients (6247 not on dialysis) randomly assigned to simvastatin 20 mg + ezetimibe 10 mg daily vs. matching placebo [23]. The reduction in both LDL cholesterol as well as the incidence of major atherosclerotic events in patients with simvastatin plus ezetimibe highlight the need for lipid lowering treatment in CKD patients. It has to be mentioned that, while hypertension and hyperlipidemia can generally be managed in primary care, other complications need to be managed in secondary care (Nephrology Clinic) by a nephrologist. Patient symptoms (ESAs for anemia) and clinical outcomes (antihypertensive agents, IV iron) improved by managing these complications [23]. We discussed the novel treatment options that have been recently introduced in the context prevention of CKD progression in type 2 diabetes, namely Glucagon-like peptide-1 (GLP-1) receptor agonist, sodium–glucose co-transporter 2 (SGLT2) inhibitors [24], and finerenone, a non-steroidal mineralocorticoid receptor antagonist [25]. George Bakris and colleagues reported the results of a study where 5734 patients with CKD and type 2 diabetes were assigned to receive finerenone (20 mg per day) or placebo. During a median follow-up of 2.6 years, a primary outcome event [kidney failure (eGFR < 15), eGFR decrease > 40%, death of renal cause] occurred in 504 of 2833 patients (17.8%) in the finerenone group and 600 of 2841 patients (21.1%) in the placebo group. So, treatment with finerenone resulted in lower risks of CKD progression and CV events than placebo. Other risk factors for CKD can be linked to environmental changes [26]. A study from Brazil [27] shows clear relationship between increasing temperatures and risk of hospitalization for CKD; there were, in fact, a total of 2,726,886 hospitalizations (females: 58.5%) due to renal diseases during 2000–2015. During the masterclass, the discussion on the impact of gender in CKD was also very thorough. The percentage of age-standardized CKD mortality has generally been lower in women (15.8%) than in men (21.6%) [28]. The data from a meta-analysis (46 cohorts from Europe, North and South America, Asia, and Australia) confirmed that the risks of all-cause mortality and cardiovascular mortality were higher in men at all levels of eGFR rate and urine albumin–creatinine (UACR) ratio [29]. A total of 261 adults (91 women and 170 men) with diabetes and CKD were spontaneously referred for nephrological evaluation by primary care physicians [30]. The main finding is that diabetic kidney disease (DKD) differs for males and females, and this difference is influenced by albuminuria and CKD progression, including smoking behaviors and low-sodium, low-phosphate, and low-potassium diets. More specifically, baseline albuminuria was lower in females [30,31]. Thus, female participants did not significantly lose eGFR during the follow-up period and the multivariate model to best predict rapid progression differed between males and females, with albuminuria not being a good biomarker of rapid progression in women. In this way, women with DKD were less frequently smokers and ingested less sodium, less phosphate and less potassium (as assessed by 24 h urinary excretions). Specifically, the dietary sodium restriction increased the anti-albuminuric effect of RAAS blockade, which was relevant for this study [31]. In women, but not in men, lower folic acid levels were a risk factor for rapid progression. Consequently, the proportion of women with predialysis CKD is higher than that of men, likely due to the longer life expectancy of women and CKD overdiagnosis with the use of eGFR equations. Kidney function declines faster in men than women, possibly owing to unhealthier lifestyles in men and the protective effects of estrogens or the damaging effects of testosterone; more men than women start RRT, not only owing to faster CKD progression in men but also because elderly women are more likely to choose conservative care. Despite the high prevalence and burden, CKD remains underdiagnosed; in fact, 5% of adults with CKD are diagnosed in stage 3, only 69% of statin-eligible adults in the US are aware that they have high cholesterol, and only the 79% of US adults with diabetes have been diagnosed [12,32]. ISN and KDIGO define patients at high risk for CKD as those with cardiovascular disease (CVD), hypertension, or diabetes. There is a high prevalence of CKD in these high-risk patients: 38%, 23%, and 36%, respectively. And yet, up to 95% of these high-risk patients are undiagnosed [33]. KDIGO guidelines recommend screening for CKD in these patients since early identification is key for improving outcomes [34]. The 2021 ESC guidelines on CVD prevention have added albuminuria to renal function for CV risk stratification [35]. New recommendations from KDIGO and ISN outline that patients with CKD stage 3a should be treated in primary care [36]. These main topics, namely complications and risk factors for CKD, were extensively discussed during the masterclass through talks from experts in the field. Moreover, during the live meetings, participants were divided into round-table groups and each group had to work and analyze a clinical task. The general aim of the round tables was to understand the perception of the mentioned health problems from young physicians and to collect suggestions on how to improve the awareness and management of CKD among the general population and nephrologists.

## 3. Hot Topics in Nephrology: The Workshops

During the workshops, participants were involved in the definition of different CKD subgroups. During the last meeting in Milan, participants were also divided into groups of work and each group prepared some slides to respond to specific ideas related to the main topics. The following hot topics were identified: (1) frailty and gender; (2) diabetes; (3) immunology and genetics; (4) hypertension and cardiovascular disease; and (5) GP awareness. The concern about CKD as a syndrome, its classification, prognostic value, and management have greatly improved during recent decades. Nephrologists now know that there are non-adaptive mechanisms for kidney loss which exist across different causes of CKD. Most of the new therapeutic options for CKD address the common pathways of CKD progression. However, there are specificities in diseases and distinctive clinical behaviors which are not considered when nephology generally mentions the term CKD. The participants were divided into groups according to their professional experience and every topic was intensely discussed group by group in the conference hall and then presented by a group leader to the faculty in a flipped classroom approach. In the following chapter, we will present the conclusions of each group.

### 3.1. Frailty and Gender

Frailty is a clinical condition in which the patient is more vulnerable to developing increased dependency and is at a higher risk of mortality when exposed to a stressor [37]. Frailty is the result of the addition of vascular, inflammatory, nutritional, and age-related insults that bring accelerated aging and lack of functional reserve [38]. It is more common in patients with CKD [39] than in patients without it [40]. In a systematic review, the prevalence of frailty was reported to range from 7% in patients with CKD stages 1–4 up to 73% in patients on hemodialysis (HD) and was associated with increased risk of mortality and hospitalization [41]. Therefore, Yang et al. [40] estimated, in a cross-sectional analysis of 177 CKD adult patients, the prevalence of frailty stratified by stage of CKD and found that the prevalence of frailty was 11.9% in stages 4–5 of CKD and 9.3% in stages 3a–3b of CKD. There are many frailty measuring tools available, such as the simple FRAIL scale [42] and the nine-point Clinical Frailty Scale [43]; however, the optimal means of screening for frailty in patients with kidney disease remains unclear. After examining the evidence in the field, the masterclass workshop highlighted the need for a multidisciplinary approach that includes education, nutrition, and psychological support. There is a difference between men and women in patients with kidney disease; in fact, the proportion of women with stage 3–5 CKD is higher than that in men, but men experience a faster progression of kidney function, observed almost globally, and are affected by kidney failure 50% more than women [44,45]. Moreover, these differences mirror a combination of physiological and social risk factors that contribute independently to CKD. The later ones influence especially women’s ability to access healthcare [46]. Also, regarding pregnancy, it is very important to perform counseling prior to gestation so the patient goes into pregnancy in a safe way, knowing the risks they face. A medical history of CKD or simply of a single episode of AKI is a risk factor for adverse pregnancy outcomes, such as preterm delivery, hypertensive disorders, or having a small-for-gestational-age baby [47,48]. On the other hand, pregnancy might be a good time to diagnose CKD in young women [49] and prevent it from progressing by detecting modifiable risk factors for future pregnancies and overall maternal health [50]. Moreover, the discovery of undiagnosed CKD can be carried out by routine laboratory tests or by the development of pre-eclampsia or other hypertensive disorders during the pregnancy [51]. Therefore, it could be useful to have joint clinics between nephrologists and obstetricians, even if only SOGC guidelines underline the importance of kidney-specific follow-up in at-risk patients [52]. Mental health issues also need to be considered, since women usually have higher rates of depression [53]. There also gender differences in kidney transplantation because of social but also biological factors: pregnancy-induced immunological responses often result in elevated levels of panel reactive antibodies in women [54]. Another significant aspect, on this topic, is transgender medicine, and more precisely the knowledge of the effects of hormone therapy on nephrology. For instance, in transgender women with CKD, estrogen should be administered with a reduction of 50% of the standard dose, as little estradiol and estrone are excreted in the urine and neither is dialyzed [55]. Estimating GFR in transgender persons is more challenging since body composition and muscle mass change because of hormone therapy with a consequential change in creatinine generation and serum creatinine [56]. The transgender population may also have additional challenges as kidney transplant recipients related to psychosocial aspects and anatomic changes after gender-affirmation surgery [57]. Moreover, hormonal therapy has its side effects: estrogen is associated with venous thromboembolism events which can lead to thrombus and allograft loss in the perisurgical phase [58], while testosterone can increase the risk of post-transplant erythrocytosis [55]. Also, the impact of different eating habits between males and females on renal functions is significant in nephrology. Finally, it would be of much interest to evaluate the impact of muscle fat infiltration between males and females affected by CKD. It is now well acknowledged that muscle abnormalities naturally occur with aging in both quality and quantity but can also be secondary to chronic disease such as CKD [59]. One of the main characteristics of malnutrition in CKD is muscle wasting [60], which can be assessed by the measurement of quadriceps muscle thickness using bedside ultrasound [61]. Interestingly, in a cross-sectional study that included 1627 Chinese adults affected by mild-to-moderate CKD, sarcopenia was significantly associated with CKD in men but not women [62].

### 3.2. Diabetes and the Kidney

Microvascular complications of diabetes include CKD, and it is commonly called diabetes-related kidney disease (DKD) [63] and defined by persistent increased proteinuria > 500 mg/24 h or a urinary albumin concentration > 300 mg/g in at least two of three urinary samples measured in an interval of 1–12 months [64]. The association of CKD with mortality has been quantified in patients with type 2 diabetes [65]. The masterclass workgroup summarized a scheme of interventions as follows. With the aim to prevent and reduce the progression of DKD, patients should be educated in lifestyle modification. First-line drug therapy with renoprotective effects include angiotensin-converting enzyme inhibitors (ACEis) and angiotensin II receptor blockers (ARBs), which reduce pressure on glomeruli by inducing efferent arteriole dilatation [66,67] and sodium–glucose transport protein 2 inhibitors (SGLT2is) that affect the tubuloglomerular feedback by increasing sodium transport to the *macula densa*, and thus reversing some of the degenerative processes associated with diabetes [68]. Large cardiovascular outcome trials have been confirmed that SGLT2is improve both cardiovascular and kidney outcomes among patients with type 2 diabetes, CKD, and eGFR ≥ 20 mL/min per 1.73 m^2^ [69,70], demonstrating not only the non-inferiority but the superiority of this drug over the standard of care. These results were confirmed by later studies in which renal endpoints were the main outcomes [71,72]. A free line of communication between primary and secondary care is required to discuss such patients. A virtual referral service where the nephrologist can comment on the patient would be a useful tool. The use of kidney biopsy in patients affected by diabetes with CKD is still controversial. The presence of unusual features, such as sudden-onset proteinuria, active urine sediment (e.g., hematuria with red cell casts) or a rapid loss in kidney function, could support the suspicion of non-diabetic nephropathy, especially in patients with a brief history of diabetes (less than five years) without retinal implication [73]. Moreover, kidney biopsies should be part of research protocols as they can help to understand the phenotype’s heterogeneity of diabetic kidney disease linked with various biomarkers, choosing a tailored targeted therapy [74]. Among lifestyle modification, the role of intermittent fasting on DKD seems to have potential benefits as has been shown in vivo studies [75,76]. Conversely, most guidelines underline that fasting over extended, longer hours increases the risk of DM complications and CKD progression primarily due to induced dehydration caused by increased perspiration and decreased fluid consumption [77]. Thus, clinical controlled trials comparing fasting CKD patients to non-fasting patients need to be started to help nephrologists, especially in the Arab world. A member of the workshop shared his clinical experience where 20% of his diabetic CKD patients are Arab Muslims that, during the month of Ramadan, are fasting between 14 and 16 h per day and they take the drug in the evening after eating so they can handle it very well.

### 3.3. Immunology and Genetics in Kidney Diseases

The masterclass work-group extensively discussed the role of immunology and genetics. Significant progress has been made in understanding the basic immune mechanisms of kidney disease and in translating these clinical findings to clinical therapies [78]. There are kidney disorders that are started and mainly mediated by an immune response, such as renal infections with reno\trophic pathogens, including mycobacterium tuberculosis and HIV; extrarenal infections with renal manifestations, including septic kidney injury, immune complex-mediated nephritis; and kidney disorders that involve renal inflammation as a secondary mechanism, such as systemic autoimmunity against ubiquitous antigens that cause renal vascular obstruction and ischemia, including scleroderma renal crisis [78]. In this workshop, the participants addressed the question of how the KDIGO classification performs as a prognostic tool [79] when compared to other disease-specific classifications and how useful it is to group different etiologies under the same CKD designation. Whether GFR and proteinuria are equally important irrespective of the cause of CKD is still unclear. As resulted in the Chronic Kidney Disease Japan Cohort study, the addition of biopsy-proven diagnoses to the GFR–albuminuria classification enhances the prediction of outcomes such as the incidence of kidney failure with replacement therapy and all-cause death [80]. Moving towards an era of precision medicine, incorporating a wider amount of individual patients’ information (such as histology and genetic tests) and artificial intelligence (AI) could guide decisions and help define prognosis [81]. AI is starting to have an impact on several aspects of nephrology: diagnosis, prognosis, and treatment [82]. For instance, for magnetic resonance imaging images, an AI system has been developed to estimate the total renal capacity of patients with ADPKD, helping clinicians in illness surveillance [83]. The participants looked into known strategies to delay CKD progression and improve cardiovascular prognosis, such as lifestyle interventions, blood pressure control, RAAS inhibition, SGLT2 inhibition, and mineralocorticoid receptor antagonists, and reviewed their role for different etiologies of kidney disease such as lupus nephritis, small vessel vasculitis, IgA nephropathy (IgAN), autosomal dominant polycystic kidney disease (ADPKD), Alport syndrome, and kidney amyloidosis. Although for rare diseases there is not enough evidence and conclusions are based on observational data, the group agreed that higher proteinuria, a rapid decline in the GFR, and uncontrolled hypertension are indeed contributors to a worse prognosis [84,85]. However, for some diseases, especially those with acute activity, the nephrologist should check if proteinuria and GFR worsening are a reflex of the acute process or the result of a chronic injury [86]. Some lifestyle interventions may be specific to some diseases (e.g., fasting, high water intake, and a ketogenic diet for ADPKD) [87,88,89] and there are specific prognostic factors (e.g., MEST-C classification for IgAN [90]; biopsy information; timely and adequate immunosuppression for lupus nephritis [91]; certain mutations for Alport syndrome [92] and ADPKD [93]; other organ involvement for amyloidosis [94]; etc.). There are also specific drugs for each disease. As for drugs aimed at altering common pathways of CKD, RAAS inhibitors and SGLT2 inhibitors have evidence across different causes of CKD, although there is a lack of evidence for rare causes. In conclusion, a personalized medicine is necessary to have tools based on histology and genomic tests for defining and stratifying CKD. It is required to try to personalize the tools that the nephrologist uses to diagnose in order to define the low or high risk of patients that need more attention. There are some phenotypes of disease, such as high proteinuria, rapid decline of the GFR, and control hypertension, that keep specific attention in order to slow the progression of the disease. It is needed to enhance the role of old and new drugs in this phenotype because the KDIGO guidelines do not provide enough scientific evidence, and it is necessary to work on observational data to use them in daily clinic practice. The bottom line is the need of a personalized approach for the treatment of CKD patients with this phenotype. In particular, there are no data on the use of SGLT2 inhibitors in rare diseases, because there are too few patients to test them, despite there being reasonable assumption that these inhibitors may be helpful in these patients.

### 3.4. Hypertension and Cardiovascular Disease Associated with Chronic Kidney Disease

Hypertension affects the great majority (60–90%) of CKD patients [51]. Hypertension and CKD are intrinsically related, as hypertension is a strong determinant of worse renal and cardiovascular outcomes and renal function decline aggravates hypertension [52]. This bidirectional relationship is well documented by the prevalence of hypertension across CKD stages and the dual benefits of effective antihypertensive drugs on renal and cardiovascular risk reduction. The success of prevention and screening programs for CKD relies on public awareness and understanding. Early detection and management are crucial to prevent disease progression. However, many CKD cases are not detected early, indicating a need for improved awareness. Populations with higher levels of knowledge about CKD show better rates of early identification and risk management. Unfortunately, research indicates inadequate awareness of CKD and its risk factors in both developed and developing nations. For instance, a study in Australia revealed insufficient awareness of the physiological importance of the kidneys, with less than half of participants correctly identifying hypertension as a risk factor [53]. The most crucial thing is to provide patients with high-quality care, which may include novel medications like SGLT2 inhibitors, GLP-1i, MRA, and lifestyle adjustments. Concerning the safety of innovative drug usage in various phenotypes, such as the continuation of SGLT-2 in a patient starting renal replacement therapy (RRT) due to heart failure or volume overload, HD/PD, or bilateral significant renal stenosis, further inquiries are required. Additionally, placing greater emphasis on certain phenotypes in the KDIGO guidelines would be suitable; patient with hypertension with a GFR3b or lower and urine anomalies are among the phenotypes that require further consideration since, even in the absence of proteinuria, a patient with hematuria can have a wide range of differential diagnoses. It is necessary to search for additional comorbidities since, if nephrologists concentrate solely on proteinuria, they may overlook other significant illnesses such as hyperparathyroidism and abnormalities of the minerals and bones. Other noteworthy phenotypes included people with diabetes with a KDIGO category of less than A3, hypertension patients with an ACR greater than 300 mg/gr on best practice guidelines for medication, and a faster fall in GFR (3–6 months by 25% in a stable condition with or without urine abnormalities). The introduction of new medications in this context can be substantiated by convincing data from well-executed clinical trials on the application of novel potassium exchangers for the treatment of hyperkalemia and the enhancement of cardiorenal therapy. This is because medications such as B blockers, RAAS inhibitors, and MRAs protect the heart and lower mortality rates in patients with this condition, and new hypolipidemic medications (inclisiran, PCSK9i) are another significant class of medications that are more effective at lowering LDL cholesterol. Additionally, GLP1RA medications may be highly beneficial for individuals with heart failure or anemia to lower inflammation or enhance lipid metabolism. It is important to remember that there is not yet clinical information about the long-term security of SGLT2 inhibitors in humans, but the same mechanism has been used in nature for millions of years. Animals that undergo estivation and need protection during very warm periods of the year, like hedgehogs, snails, and some birds, go into dormancy to protect them during these hot periods and the changes in their hepatorenal metabolism are almost exactly the same as we have with SGLT2 inhibitors, so the same mechanism has been working in nature for millions of years and, now, in the 21st century, the nephrologist must have learned how to manipulate this. Regarding adherence to treatment, the best possible adherence is up to 50% in renal patients, where the treatment usually includes over 10 drugs; the adherence is generally very poor in this phenotype, so when thinking about hypertension treatment, the nephrologist has to focus at first on all the long acting drugs and prescribing as few pills as possible, on combo drugs, and considering hypervolemia. Therefore, in the case of the resistant hypertension dialysis population, it is possible to start with bio-impedance to look what is hypervolemia and, if it is between 5 and 10 L, it is possible to start with a different dialysis prescription and not with escalation of a drug dose.

### 3.5. Chronic Kidney Disease Awareness in General Practice

In this paper, attention has been paid to the distinction between reality, where there are no international standards for screening, but there is an underdiagnosis of CKD, and an ideal scenario, where the risk groups, such as diabetic patients, hypertensive patients, or patients with CV disease, that could benefit from screening are be defined.

More than 850 million people worldwide have CKD, and by 2040 CKD is predicted to be the fifth most prevalent chronic condition in the world. Although being highly prevalent, only one-third of patients are diagnosed with CKD due to its typically asymptomatic nature in the early stages and the absence of a systematic screening approach. Furthermore, CKD is typically regarded as a consequence of diabetes or hypertension, rather than as an independent disease, thereby postponing referral to a nephrologist until the condition has reached late stages [95,96,97]. KDIGO guidelines define CKD as a decrease in the estimated glomerular filtration rate (eGFR) (<60 mL/min per 1.73 m^2^) and albuminuria for a duration of at least 3 months. However, the first typical diagnostic indicator is a decreased eGFR, and the use of the eGFR CKD-EPI formula is recommended. The World Health Organization (WHO) mandates screening for diseases that present a significant public health concern, and CKD fulfills these requirements. Recently, attention has moved from dialysis to the prevention of ESKD due to the use of drugs such as SGLT2 inhibitors. Increased awareness and precise identification of the groups at risk to be examined, including those with diabetes, hypertension, cardiovascular disorders, and obesity, are crucial for enhancing early detection (Figure 1).

It is needed to increase the awareness about this phenotype in our countries, because in reality there is a big awareness gap among the population on how our bodies and kidneys work. Many people do not know what to drink and how much to drink. CKD treatments are also probably too complicated because there are many pills to take and because the adherence to the treatment decreases significantly with time. So, the explanation of how the kidneys work to the general population would be an ideal setting. The importance of CKD should be emphasized in medical school; it is necessary to make nephrology popular and to somehow raise the awareness and attention between students. Regarding the interaction between General Practitioners (GPs) and nephrologists, it is needed to build a bridge; for invitations to nephrology congresses, the information should be made into leaflets so that it will be easier for them to diagnose and treat CKD patients. On this topic, the multidisciplinary approach is significant so that complications of CKD can be addressed promptly. The ideal situation could be the development of practical recommendations on CKD management for GPs, including the drugs, the diet, and the hygienic regimen of these patients. These can include explanations of the diet, what exactly to eat and drink with specific recipes for patients initiating and titrating the drugs in order to optimize the dosage, and inviting patients to not evaluate creatinine immediately after SGLT2 inhibitor initiation because the patient will become frightened by the slight increase in the creatinine and will stop the medication. A critical point for GPs is the age problem. In our opinion, forcing GPs may become necessary or also using an age set by nephrologists, as urologists do for PSA, and to require this also after creatinine. Another important suggestion to provide during a potential discussion with politicians is that CKD can be seen as a barometer of planetary health, so the kidneys are of extreme importance for our internal milieu because they are influenced by air pollution, food, heat, stress, and lack of water. In addition, it is required by the panel to simplify the CKD risk factors (e.g., obesity, age, and smoking) for GPs. It could be a good idea to talk about dialysis in the first nephrology consult, but doctors need to know how to communicate this and to choose correlated words. The nephrologist should also ask themself how GPs can help them, or, the other way around, not what the nephrologists are expecting but what they can obtain from the other side. This is the first point on how to build the bridge between nephrologists and GPs. In conclusion, dialog with patient (with numbers and graphs) is also a nice tool when talking with the patient about renal replacement therapy and health risks.

**Figure 2:**
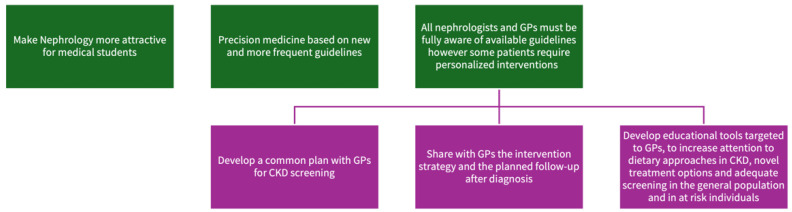
Key messages of the discussed topic.

Some strategies to impact awareness between GPs and other physicians can be the following: (1) Educational Campaigns: organizing webinars, workshops, and conferences on CKD, risk factors, and early signs, in partnership with medical associations to disseminate educational materials; (2) Clinical Guidelines and Decision Support: make the updated guidelines on CKD accessible and provide tools to facilitate diagnosis and management in daily practice; (3) Continuing Medical Education (CME): to implement CME programs on CKD for physicians, including credits or certifications and case-based learning; (4) Integration into Primary Care Practice: to encourage the integration of CKD screening and management into routine primary care practices, especially for patients with risk factors such as diabetes and hypertension; (5) Patient Education Materials: to develop patient-friendly educational materials that GPs can share with their patients, to reinforce awareness about kidney health; (6) Collaboration with Nephrologists: to facilitate collaboration and regular communication between GPs and nephrologists for complex cases; (7) Digital Tools and Technology: To leverage digital platforms to disseminate information and to develop apps, online modules, or podcasts that physicians can access, implementing electronic health record (EHR) prompts to remind doctors about the importance of kidney function testing for at-risk patients; (8) Quality Improvement Initiatives: to implement programs that monitor and enhance CKD management in primary care, rewarding exemplary methods; (9) Advocacy and Policy Initiatives: to advocate for policies that support CKD awareness and screening in primary care. This may include reimbursement incentives or requirements for routine screening; (10) Research and Data Sharing: to support and conduct research on CKD prevalence and outcomes in primary care settings. One should foster a culture of data sharing and collaboration among healthcare professionals to enhance collective knowledge. A summary of the main key points of this paper is reported in Figure 2.

## 4. Conclusions

In conclusion, all the participants confirmed that the masterclass was a clearly focused meeting giving an overview of the key advancements in nephology, as well as a forum where experts from around the world were able to meet, understand different clinical healthcare systems, exchange ideas and start collaborations, and, just as importantly, to have fun while doing so. This event was of exceptional importance in the field of nephrology and allowed a collaborative approach between colleagues from different countries and professional backgrounds. The rich variety of speakers provided fresh impetus for a nephrological update. We have tried to provide learning opportunities from experts, but also, we invited many international colleagues to actively participate in sharing their clinical experience and research, with the common aim to improve the quality of the treatment of CKD patients.

## Data Availability

Not applicable.
